# Neutralizing activity of anti–SARS-CoV-2 hyperimmune immunoglobulins and intravenous immunoglobulins against currently circulating SARS-CoV-2 variants

**DOI:** 10.1172/JCI182919

**Published:** 2024-07-25

**Authors:** Lorenza Bellusci, Hana Golding, Surender Khurana

**Affiliations:** Division of Viral Products, Center for Biologics Evaluation and Research (CBER), FDA, Silver Spring, Maryland, USA.

**Keywords:** Infectious disease, Therapeutics, Clinical practice, Immunoglobulins, Immunotherapy

**To the Editor:** Prophylactic or early post-exposure treatments with SARS-CoV-2–specific monoclonal antibodies (mAbs) were useful early in the COVID-19 pandemic. However, the currently circulating SARS-CoV-2 Omicron subvariants (e.g., XBB.1, JN.1 and its derivatives) are resistant to all approved mAb therapies (1). Immunoglobulin products (IGs) manufactured from pooled human plasma are widely used for treatment of patients with several immunodeficiency syndromes. Most IGs are administered intravenously and are called IVIGs.

Polyclonal hyperimmune anti–SARS-CoV-2 IVIGs (pi-hCoV-2IG) were manufactured in 2021 by fractionation of pooled plasma from COVID-19 convalescent patients with virus neutralization titers of 1:320 or greater against the ancestral WA-1 strain and contain IgG at 10-fold higher concentration than in individual convalescent plasma (CP). Vx-hCoV-2IG was generated from pooled plasma of SARS-CoV-2–vaccinated individuals (2021) (2). Some vaccinated individuals also reported prior SARS-CoV-2 infection. Since 2022, more than 90% of the blood donations in the United States had anti–SARS-CoV-2 antibodies, suggesting prior exposure by vaccination, infections, or both (hybrid immunity) (3). Therefore, we hypothesized that IVIG lots manufactured from unscreened plasma donors from 2022 onwards may contain anti–SARS-CoV-2 neutralizing antibodies against circulating Omicron subvariants.

To evaluate therapeutic potential of multiple lots of IVIG, pi-hCoV-2IG, and Vx-hCoV-2IG against circulating Omicron variants (Supplemental Table 1; supplemental material available online with this article; https://doi.org/10.1172/JCI182919DS1), we followed the STROBE reporting guideline (https://www.strobe-statement.org/) for cross-sectional studies. We tested 17 lots of pi-hCoV-2IG prepared from pooled plasma of convalescent individuals infected with SARS-CoV-2 in 2020 and one available Vx-hCoV-2IG lot manufactured from screened pooled plasma with high SARS-CoV-2 neutralization titers of mRNA-vaccinated individuals (hybrid immunity) who reported prior SARS-CoV-2 infection in 2021. Additionally, 20 IVIG preparations manufactured in 2019 from healthy plasma donations (2019-IVIG) before the COVID-19 pandemic, 8 IVIG lots manufactured in 2020 (2020-IVIG), 9 IVIG lots manufactured in 2023 (2023-IVIG), 5 IVIG lots manufactured in 2024 (2024-IVIG), 7 CP from recovered COVID-19 patients in early 2020 (2020-CP), and 8 CP from Omicron vaccine breakthrough infections in 2022 (2022-CP), all collected approximately 30 days after diagnosis, were analyzed for neutralization of SARS-CoV-2 WA-1 and 9 circulating Omicron subvariants (BA.2.86, XBB.1.16, XBB.2.3, EG.5, HV.1, HK.3, JN.1, JN.4, and JD.1.1) in a pseudovirus neutralization assay (PsVNA) (4).

CP collected from recovered COVID-19 patients in 2020 and 2022 as well post-infection hyperimmunoglobulin lots (pi-hCoV-2IG) show high neutralization titers against WA-1, but demonstrated minimal or no PsVNA titers against the current Omicron variants (Figure 1). The 2019-IVIG lots manufactured before the COVID-19 pandemic contained no SARS-CoV-2–neutralizing antibodies (Figure 1). The 2020-IVIG lots manufactured early in the COVID-19 pandemic contained low titers against WA-1, and no neutralizing titers against current Omicron subvariants. On the other hand, 2023-IVIG and 2024-IVIG lots contain high titers against WA-1 (geometric mean titer [GMT]: 16,212 and 30,722, respectively), reflecting the high SARS-CoV-2 seroprevalence in plasma donors. More importantly, these recent IVIG lots have weak to moderate titers against currently circulating variants, even though the donors were exposed to earlier Omicron variants (Supplemental Table 2). The surprising finding was that one lot of Vx-hCov-2IG that was produced in 2021 not only contains the highest titer against the original WA-1 strain (GMT: 69,551), but also high neutralization titers against all the recently circulating Omicron subvariants (GMT ranging between 401 and 11,416) (Figure 1). This finding may be due to vaccination/infection-induced cross-reactive B cells undergoing affinity maturation in germinal centers, resulting in broader high-affinity antibody repertoires that neutralize emerging Omicron subvariants (5).

Our finding demonstrates that high-titer IVIG lots can be manufactured from plasma screened for high neutralization titers against recent Omicron subvariants. Since the current circulating SARS-CoV-2 Omicron subvariants are resistant to all licensed mAbs, these cross-neutralizing IVIG lots can be an important intervention, particularly for immunocompromised patients and various autoimmune and neurological diseases, including patients with long COVID (6), to prevent or ameliorate the outcome of exposure with circulating and emerging SARS-CoV-2 strains.

## Supplementary Material

Supplemental data

Supporting data values

## Figures and Tables

**Figure 1:**
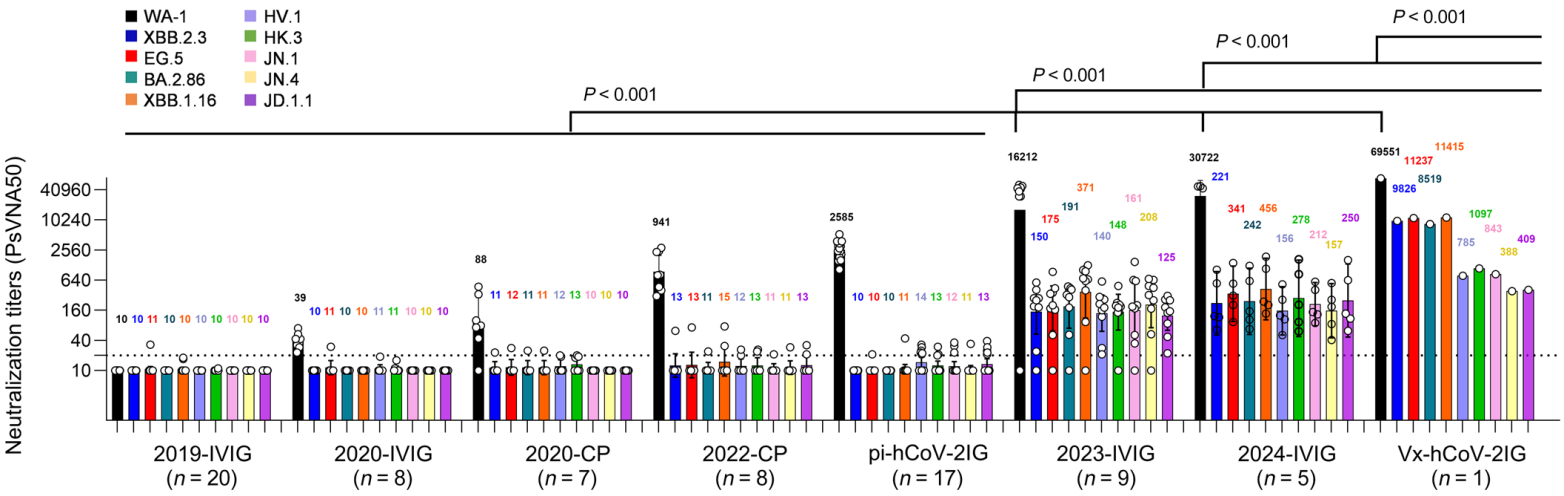
Neutralization of SARS-CoV-2 WA1/2020 and circulating Omicron subvariants by IVIG, convalescent plasma, pi-hCoV-2IG, and Vx-hCoV-2IG. SARS-CoV-2 neutralization assays were performed by using pseudoviruses expressing the spike protein of WA1/2020 or the Omicron subvariants in 293-ACE2-TMPRSS2 cells. SARS-CoV-2 neutralization titers were determined in each of the prepandemic 2019-IVIG (*n* = 20), 2020-IVIG (*n* = 8), 2020 convalescent plasma (2020-CP; *n* = 7), 2022 convalescent plasma (2022-CP; *n* = 8), post-infection hyperimmunoglobulin IVIG (pi-hCoV-2IG; *n* = 17), 2023-IVIG (*n* = 9), 2024-IVIG (*n* = 5), and post-vaccination hyperimmunoglobulin IVIG (Vx-hCoV-2IG; *n* = 1) preparations. The assay was performed in duplicate to determine the 50% neutralization titer (PsVNA50). The heights of the bars and the numbers over the bars indicate the geometric mean titers, and the whiskers indicate 95% confidence intervals. The horizontal dashed line indicates the limit of detection for the neutralization assay (PsVNA50 of 20). Differences between SARS-CoV-2 strains were analyzed by ordinary 1-way ANOVA with Tukey’s pairwise multiple-comparison test in GraphPad Prism version 9.3.1 and the *P* values are shown.

## Version 1 10/15/2024

Electronic publication
