# Supplementary material for: Neutralizing activity of anti–SARS-CoV-2 hyperimmune immunoglobulins and intravenous immunoglobulins against currently circulating SARS-CoV-2 variants
Source: J Clin Invest. 2024 Jul 25;134(20):e182919. doi: 10.1172/JCI182919 (PMC11473157; doi:10.1172/JCI182919)
Supplement: Supplemental data [file jci-134-182919-s209.pdf]

SUPPLEMENTAL APPENDIX

Supplement to:

Neutralizing activity of SARS-CoV-2 hyperimmune immunoglobulins and intravenous immunoglobulins against currently circulating SARS-CoV-2 variants

by Lorenza Bellusci, Hana Golding and Surender Khurana.

TABLE OF CONTENTS

CONTENTS

1. Materials and Methods
2. Table S1. SARS-CoV-2 variants mutations introduced in the spike plasmid for production of SARS-CoV-2 pseudovirions for analysis in PsVNA
3. Table S2. Summary of CP and IVIG lots and neutralization titers
4. Acknowledgments
5. Supplementary References

MATERIALS AND METHODS

Sex as a biological variable

Sex was not considered as a biological variable. IVIG lots were purchased from commercial manufactures who manufactured it from pooled plasma from 200-1000s of donors. Seven random CP lots were obtained from recovered COVID-19 patients between May-September 2020. Sex information was not provided by the supplier.

Samples and study design

IVIG products approved in the United States are polyclonal antibody preparations made from 10,000 or more U.S. plasma donors and may include cold alcohol fractionation (Cohn-Oncley), anion-exchange and size-exclusion chromatography's. The final product is sterile-filtered IgG (>95%) and formulated at 100 mg/mL. Twenty intravenous immunoglobulin batches were produced from plasma collected prior to August 2019 (2019-IVIG) and eight IVIG lots made from plasma donations in 2020 (2020-IVIG) and manufactured between October 2020 and January 2021 (each lot derived from >10,000 donors), were obtained from six manufacturers.

Seventeen pi-hCoV-2IG batches prepared from post-SARS-CoV-2 infection CP collected at least 30-days post-recovery (~200-1000 US plasma donors per lot) were obtained/purchased from four commercial companies for blinded antibody analysis. The plasma units used in the manufacturing of the hCoV-2IG batches were collected in 2020 (during circulation of ancestral Wuhan, D614G or alpha strains) prior to emergence of the Delta and Omicron VOCs or prior to availability of COVID-19 vaccines. Nine IVIG lots manufactured in 2023 (2023-IVIG) and five IVIG lots manufactured in 2024 (2024-IVIG) were obtained from four manufacturers. One hyperimmune intravenous immunoglobulin lot (Vx-hCoV-2IG) produced from plasma collected

from SARS-CoV-2 vaccinated individuals at least 2 weeks after second vaccination (most with prior COVID-19 infection) in 2021 (during the alpha and delta circulation) prior to circulation of Omicron was obtained from one manufacturer.

Seven random CP lots were obtained from recovered COVID-19 patients between May-September 2020 (at least 30-days post-recovery) prior to COVID-19 vaccinations. At the time of collection SARS-CoV-2 D614G was the predominant strain in the US. Eight CP lots were collected in February 2022 from recovered individuals following Omicron breakthrough infections (probably BA.1), who received at least two doses of COVID-mRNA vaccination.

Neutralization assay

Samples were evaluated in a qualified SARS-CoV-2 pseudovirion neutralization assay (PsVNA) using SARS-CoV-2 WA1/2020 strain and circulating Omicron subvariants: BA.2.86, XBB.1.16, XBB.2.3, EG.5, HV.1, HK.3, JN.1, JN.4 and JD.1.1. The mutations in spike protein of these Omicron subvariants are shown in Supplementary Table S1. SARS-CoV-2 neutralizing activity measured by PsVNA correlates with PRNT (plaque reduction neutralization test with authentic SARS-CoV-2 virus) in previous studies (1-3). However, some antibodies targeting the N-terminal domain of SARS-CoV-2 spike may not show neutralization in the pseudovirus neutralization assay.

Neutralization assays were performed as previously described (2, 4). Briefly, 50 µL of SARS-CoV-2 S pseudovirions (counting ~200,000 relative light units) were pre-incubated with an equal volume of medium containing serial dilutions (starting at 1:10) of all samples at room temperature for 1 h. Then, 50 µL of virus-antibody mixtures were added to 293T-ACE2-

TMPRSS2 cells [10^4 cells/50 µL; gift from the laboratory of Carol Weiss (1)] in a 96-well plate. The input virus with all SARS-CoV-2 strains was the same (2 x 10^5 relative light units/50 µL/well). After a 3 h incubation, fresh medium was added to the wells. Cells were lysed 24 h later, and luciferase activity was measured using One-Glo luciferase assay system (Promega). The assay of each sample was performed in duplicate, and the 50% neutralization titer was calculated using Prism 9 (GraphPad Software). The limit of detection for the neutralization assay is 1:20. Two independent biological replicate experiments were performed for each sample and variation in PsVNA50 titers was <10% between replicates.

Quantification and statistical analysis

Descriptive statistics were performed to determine the geometric mean titer values and were calculated using GraphPad. All experimental data to compare differences among groups were analyzed using Ordinary one-way ANOVA with Tukey's pairwise multiple comparison test in GraphPad Prism version 9.3.1. To ensure robustness of the results, absolute measurements were log2-transformed before performing the analysis.

Study approval

This study was reviewed and approved by the Food and Drug Administration's Research Involving Human Subjects Committee (RIHSC #2020-04-02). This study complied with all relevant ethical regulations for work with human participants, and written informed consent was obtained. Samples were collected from adult subjects who provided informed consent to

participate in the study. All assays performed fell within the permissible usages in the original informed consent.

Data availability

All underlying data shown in the manuscript is provided in Supplementary Table S2 of the supplementary appendix.

Supplementary Table S1: SARS-CoV-2 variants mutations introduced in the spike plasmid for production of SARS-CoV-2 pseudovirions for analysis in PsVNA.

SARS-CoV-2 variant	Mutations constructed in the spike plasmids
Omicron (BA.2.86)	BA.2 spike mutations (T19I, delL24, delP25, delP26, A27S, G142D, V213G, G252V, G339D, S371F, S373P, S375F, T376A, D405N, R408S, K417N, N440K, S477N, T478K, E484A, Q493R, Q498R, N501Y, Y505H, D614G, H655Y, N679K, P681H, N764K, D796Y, Q954H, N969K) + D339H, K356T, A570V, V445H, R493Q, F486P
Omicron (XBB.1.16)	XBB.1.5 spike mutations (T19I, delL24, delP25, delP26, A27S, G142D, V213G, G252V, G339D, S371F, S373P, S375F, T376A, D405N, R408S, K417N, N440K, S477N, T478K, E484A, Q493R, Q498R, N501Y, Y505H, D614G, H655Y, N679K, P681H, N764K, D796Y, Q954H, N969K, V83A, Del144, H146Q, Q183E, V213E, G339H, R346T, L368I, V445P, G446S, N460K, F486P, F490S, R493Q) + E180V, K478R
Omicron (XBB.2.3)	XBB.1 spike mutations (T19I, delL24, delP25, delP26, A27S, G142D, V213G, G252V, G339D, S371F, S373P, S375F, T376A, D405N, R408S, K417N, N440K, S477N, T478K, E484A, Q493R, Q498R, N501Y, Y505H, D614G, H655Y, N679K, P681H, N764K, D796Y, Q954H, N969K, V83A, Del144, H146Q, Q183E, V213E, G339H, R346T, L368I, V445P, G446S, N460K, F486S, F490S, R493Q) + D253G, P521S
Omicron (EG.5)	XBB.1.5 spike mutations (T19I, delL24, delP25, delP26, A27S, G142D, V213G, G252V, G339D, S371F, S373P, S375F, T376A, D405N, R408S, K417N, N440K, S477N, T478K, E484A, Q493R, Q498R, N501Y, Y505H, D614G, H655Y, N679K, P681H, N764K, D796Y, Q954H, N969K, V83A, Del144, H146Q, Q183E, V213E, G339H, R346T, L368I, V445P, G446S, N460K, F486P, F490S, R493Q) + Q52H, F456L
Omicron (HV.1)	EG.5 spike mutations (T19I, delL24, delP25, delP26, A27S, G142D, V213G, G252V, G339D, S371F, S373P, S375F, T376A, D405N, R408S, K417N, N440K, S477N, T478K, E484A, Q493R, Q498R, N501Y, Y505H, D614G, H655Y, N679K, P681H, N764K, D796Y, Q954H, N969K, V83A, Del144, H146Q, Q183E, V213E, G339H, R346T, L368I, V445P, G446S, N460K, F486P, F490S, R493Q, Q52H, F456L) + L452R, F1527L
Omicron (HK.3)	EG.5 spike mutations (T19I, delL24, delP25, delP26, A27S, G142D, V213G, G252V, G339D, S371F, S373P, S375F, T376A, D405N, R408S, K417N, N440K, S477N, T478K, E484A, Q493R, Q498R, N501Y, Y505H, D614G, H655Y, N679K, P681H, N764K, D796Y, Q954H, N969K, V83A, Del144, H146Q, Q183E, V213E, G339H, R346T, L368I, V445P, G446S, N460K, F486P, F490S, R493Q, Q52H, F456L) + L455F
Omicron (JN.1)	BA.2.86 mutations (T19I, delL24, delP25, delP26, A27S, G142D, V213G, G252V, G339D, S371F, S373P, S375F, T376A, D405N, R408S, K417N, N440K, S477N, T478K, E484A, Q493R, Q498R, N501Y, Y505H, D614G, H655Y, N679K, P681H, N764K, D796Y, Q954H, N969K, D339H, K356T, A570V, V445H, R493Q, F486P) + L455S
Omicron (JN.4)	BA.2.86 mutations (T19I, delL24, delP25, delP26, A27S, G142D, V213G, G252V, G339D, S371F, S373P, S375F, T376A, D405N, R408S, K417N, N440K, S477N, T478K, E484A, Q493R, Q498R, N501Y, Y505H, D614G, H655Y, N679K, P681H, N764K, D796Y, Q954H, N969K, D339H, K356T, A570V, V445H, R493Q, F486P, L455S) + A475V
Omicron (JD.1.1)	XBB.1.5 spike mutations (T19I, delL24, delP25, delP26, A27S, G142D, V213G, G252V, G339D, S371F, S373P, S375F, T376A, D405N, R408S, K417N, N440K, S477N, T478K, E484A, Q493R, Q498R, N501Y, Y505H, D614G, H655Y, N679K, P681H, N764K, D796Y, Q954H, N969K, V83A, Del144, H146Q, Q183E, V213E, G339H, R346T, L368I, V445P, G446S, N460K, F486P, F490S, R493Q) + L455F, F456L, A475V

Supplementary Table S2: Neutralization titers of convalescent plasma, IVIG and hCoV-2IG against SARS-CoV-2 variants*

	WA-1	XBB.2.3	EG.5	BA.2.86	XBB.1.16	HV.1	HK.3	JN.1	JN.4	JD.1.1
**IVIG batches produced in 2019 prior to COVID-19**										
2019-IVIG-1	10	10	10	10	10	10	10	10	10	10
2019-IVIG-2	10	10	10	10	10	10	10	10	10	10
2019-IVIG-3	10	10	10	10	10	10	10	10	10	10
2019-IVIG-4	10	10	10	10	33	10	10	10	10	10
2019-IVIG-5	10	10	10	10	10	10	10	10	10	10
2019-IVIG-6	10	10	10	10	10	10	10	10	10	10
2019-IVIG-7	10	10	10	10	10	10	10	10	10	10
2019-IVIG-8	10	10	10	10	10	10	10	10	10	10
2019-IVIG-9	10	10	10	10	10	10	10	10	10	10
2019-IVIG-10	10	10	10	10	10	10	10	10	10	10
2019-IVIG-11	10	10	10	10	10	10	10	10	10	10
2019-IVIG-12	10	10	10	17	10	10	10	10	10	10
2019-IVIG-13	10	10	10	10	10	10	10	10	10	10
2019-IVIG-14	10	10	10	18	10	10	10	10	10	10
2019-IVIG-15	10	10	10	10	10	10	10	10	10	10
2019-IVIG-16	10	10	10	10	10	10	10	10	10	10
2019-IVIG-17	10	10	10	10	10	10	10	10	10	10
2019-IVIG-18	10	10	10	10	10	10	10	10	11	10
2019-IVIG-19	10	10	10	10	10	10	10	10	10	10
2019-IVIG-20	10	10	10	10	10	10	10	10	10	10
**IVIG batches produced in 2020 (circulating SARS-CoV-2 strains: Wuhan, D614G, and Alpha)**										
2020-IVIG-1	36	10	10	10	10	10	10	10	10	10
2020-IVIG-2	32	10	10	10	10	10	10	10	11	10
2020-IVIG-3	70	10	10	10	30	10	10	10	10	10
2020-IVIG-4	43	10	10	10	10	10	10	10	16	10
2020-IVIG-5	53	10	10	10	10	10	10	10	10	10
2020-IVIG-6	43	10	10	10	10	10	10	10	10	10
2020-IVIG-7	29	10	10	10	10	10	10	10	10	10
2020-IVIG-8	23	10	10	10	10	10	10	19	10	10
**Convalescent plasma batches produced from COVID-19 survivors collected in 2020 (circulating SARS-CoV-2 strains: Wuhan, D614G, and Alpha)**										
2020-CP-1	333	10	10	10	10	10	10	19	10	10
2020-CP-2	101	10	10	10	10	10	10	10	10	10
2020-CP-3	10	10	10	10	10	10	10	10	18	10
2020-CP-4	79	10	10	10	10	10	10	10	10	10
2020-CP-5	44	23	25	25	28	10	10	10	20	10
2020-CP-6	73	10	10	10	10	10	10	20	19	10
2020-CP-7	473	10	10	10	10	10	10	10	10	10
**Convalescent plasma batches produced from COVID-19 survivors collected in 2022 (circulating SARS-CoV-2 strains: Omicron BA.1 and BA.2)**										
2022-CP-1	2419	62	24	76	73	29	21	20	26	21

2022-CP-2	827	10	10	10	10	10	10	10	10	10
2022-CP-3	307	10	10	10	10	10	10	10	10	10
2022-CP-4	399	10	10	10	10	10	10	10	10	10
2022-CP-5	802	10	10	10	10	10	10	10	10	10
2022-CP-6	2858	10	10	10	10	10	10	10	10	10
2022-CP-7	2335	10	10	31	10	10	10	26	24	32
2022-CP-8	469	10	10	10	10	10	10	10	10	10
**pi-hCoV-2IG batches produced from COVID-19 donors (circulating SARS-CoV-2 strains: Wuhan, D614G, and Alpha)**										
pi-hCoV-2IG-1	3064	10	10	10	21	34	10	33	30	39
pi-hCoV-2IG-2	2102	10	10	10	10	10	10	29	21	21
pi-hCoV-2IG-3	3459	10	10	10	10	10	31	25	10	10
pi-hCoV-2IG-4	1555	10	10	10	10	10	36	10	10	10
pi-hCoV-2IG-5	1047	10	10	10	10	10	10	10	10	10
pi-hCoV-2IG-6	2050	10	10	10	10	10	10	10	10	10
pi-hCoV-2IG-7	1836	10	10	10	10	10	10	10	10	10
pi-hCoV-2IG-8	2438	10	10	10	10	10	10	10	10	10
pi-hCoV-2IG-9	2452	10	10	10	10	10	10	10	10	10
pi-hCoV-2IG-10	2977	10	10	44	10	10	10	10	20	10
pi-hCoV-2IG-11	3522	10	10	10	10	10	10	23	20	25
pi-hCoV-2IG-12	2777	10	10	10	10	10	23	10	10	10
pi-hCoV-2IG-13	1602	10	10	10	10	10	10	10	10	10
pi-hCoV-2IG-14	3472	10	10	10	10	10	10	21	10	10
pi-hCoV-2IG-15	3834	10	10	10	10	10	10	22	10	10
pi-hCoV-2IG-16	5285	10	10	10	10	10	10	21	21	29
pi-hCoV-2IG-17	3825	10	10	10	10	10	10	10	10	23
**IVIG batches produced in 2023 (circulating SARS-CoV-2 strains: Omicron XBB.1.5, XBB.1.16, XBB.2.3, EG.5, BA.2.86, JN.1, JN.4, HK.3, HV.1, JD.1.1)**										
2023-IVIG-1	30201	24	52	94	51	53	51	28	148	47
2023-IVIG-2	42551	192	181	661	175	173	35	119	144	87
2023-IVIG-3	10	10	10	10	10	10	10	21	10	22
2023-IVIG-4	51810	278	420	401	164	254	1488	160	225	183
2023-IVIG-5	49281	573	478	1266	940	646	651	600	493	264
2023-IVIG-6	45821	408	441	1019	361	322	618	263	190	311
2023-IVIG-7	44281	279	415	963	443	489	201	295	207	143
2023-IVIG-8	30452	183	326	614	342	681	175	215	196	255
2023-IVIG-9	38161	255	294	693	202	452	191	182	182	153
**IVIG batches produced in 2024 (circulating SARS-CoV-2 strains:KP.1, JN.1, JN.1.7)**										
2024-IVIG-1	43925	116	104	240	93	85	147	105	93	110
2024-IVIG-2	16768	122	153	205	314	187	133	126	208	137
2024-IVIG-3	47724	1037	1240	1087	1420	560	597	260	1680	1624
2024-IVIG-4	15980	65	68	138	204	41	89	51	61	62
2024-IVIG-5	48724	555	614	1896	547	260	408	525	841	640
**Vx-hCoV-2IG batches produced from SARS-CoV-2 vaccinated plasma donors (mRNA vaccinated US plasma donors with prior COVID-19 with either Alpha or Delta variants)**										

Vx-hCoV-2IG	69551	9826	8519	11415	11237	388	843	785	1097	409

* PsVNA titer Cut-off value: 1:10.

Author contributions

SK and HG designed the research. HG provided clinical specimens and unblinded the clinical data. LB and SK performed assays. SK and HG contributed to writing the manuscript. SK had full access to all the data in the study and takes responsibility for the integrity of the data and the accuracy of the data analysis.

Acknowledgments

We thank Basil Golding and Keith Peden at FDA for review of the manuscript. We thank Carol Weiss (FDA) for providing plasmid clones expressing SARS-CoV-2 spike variants.

Supplementary References

1. Neerukonda SN, Vassell R, Herrup R, Liu S, Wang T, Takeda K, et al. Establishment of a well-characterized SARS-CoV-2 lentiviral pseudovirus neutralization assay using 293T cells with stable expression of ACE2 and TMPRSS2. *PLoS One.* 2021;16(3):e0248348.
2. Tang J, Lee Y, Ravichandran S, Grubbs G, Huang C, Stauft CB, et al. Epitope diversity of SARS-CoV-2 hyperimmune intravenous human immunoglobulins and neutralization of variants of concern. *iScience.* 2021:103006.
3. Ravichandran S, Coyle EM, Klenow L, Tang J, Grubbs G, Liu S, et al. Antibody signature induced by SARS-CoV-2 spike protein immunogens in rabbits. *Sci Transl Med.* 2020;12(550):eabc3539.
4. Ravichandran S, Lee Y, Grubbs G, Coyle EM, Klenow L, Akasaka O, et al. Longitudinal antibody repertoire in "mild" versus "severe" COVID-19 patients reveals immune markers associated with disease severity and resolution. *Sci Adv.* 2021;7(10).
